# Supplementary material for: Temperature overshoot responses to ambitious forestation in an Earth System Model
Source: Nat Commun. 2024 Sep 19;15:8235. doi: 10.1038/s41467-024-52508-x (PMC11413198; doi:10.1038/s41467-024-52508-x)
Supplement: Supplementary file 1 — Supplementary Information [file 41467_2024_52508_MOESM1_ESM.pdf]

Supplementary Information for: Temperature overshoot responses to ambitious forestation in an Earth System Model

Yiannis Moustakis[1]*, Tobias Nützel[1], Hao-Wei Wey[2], Wenkai Bao[1], Julia Pongratz[1,3]

[1] Ludwig-Maximilians-Universität in Munich, Munich, Germany

[2] GEOMAR Helmholtz Centre for Ocean Research Kiel, Kiel, Germany

[3] Max Planck Institute for Meteorology, Hamburg, Germany

*corresponding author, email: yiannis.moustakis@geographie.uni-muenchen.de

Supplementary figures

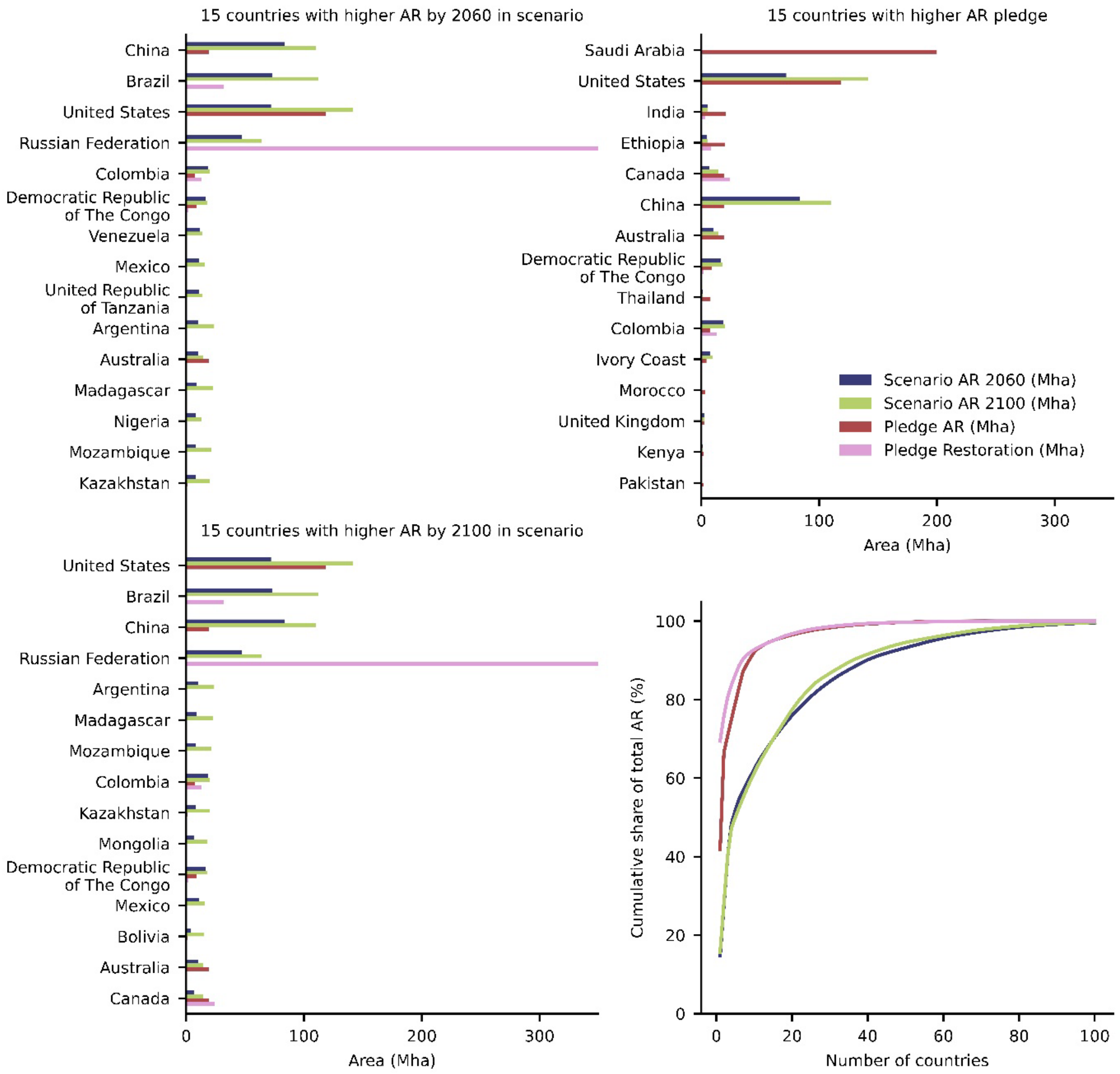

Supplementary Figure 1: Country level AR data: The bar plots show country level data of land-based mitigation pledges for AR and ecosystem restoration [1,2], and the amount of AR achieved by 2060 and 2100 in the scenario employed in this study. The three plots show (top left) the 15 countries with the higher amount of AR reached in 2060 in our scenario, (top right) the 15 countries with the higher AR pledge based on the Land-Gap report estimates [1,2], and (bottom left) the 15 countries with the higher amount of AR reached in 2100 in our scenario. For these quantities, the cumulative share (%) with increasing number of countries sorted in descending order is shown (bottom right).

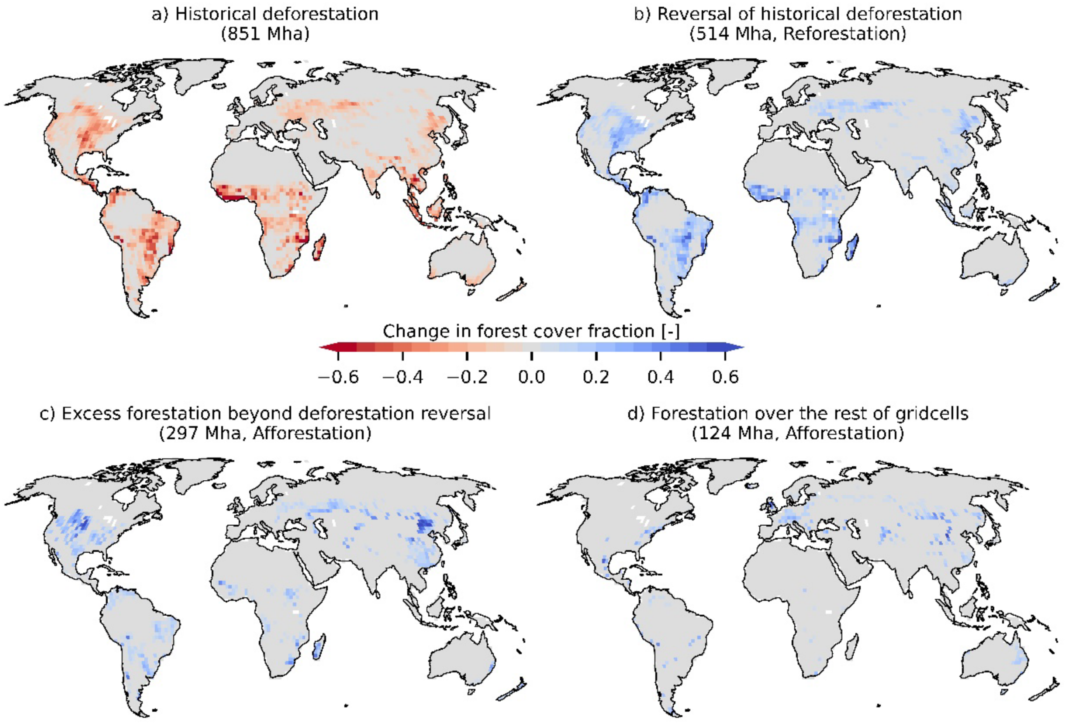

Supplementary Figure 2: Features of the AR pattern: This figure shows (a) the historical deforestation pattern in MPI-ESM, (b) the reversal of historical deforestation through AR in our scenario, (c) afforestation over gridcells where historical deforestation occurred, increasing forest cover beyond 1850 levels, and (d) afforestation over gridcells where historical deforestation has not occurred. Changes are expressed as changes in forest cover fraction at the gridcell level.

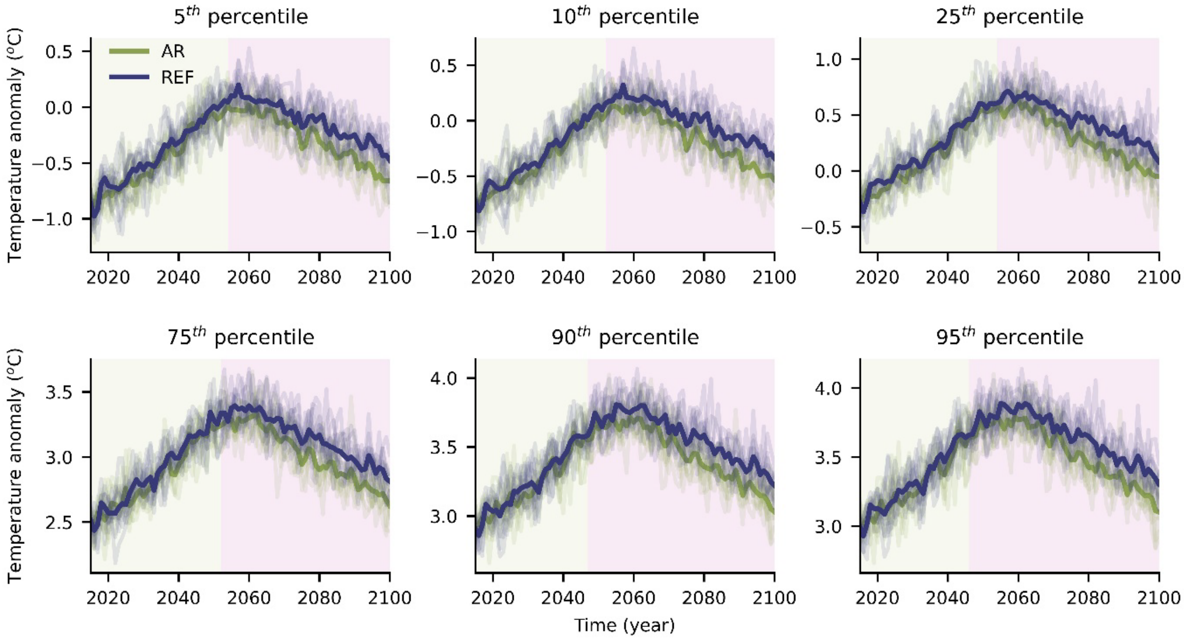

Supplementary Figure 3: The panels show different percentiles of 2m air temperature (difference compared to pre-industrial era, expressed here as the 1850-1900 average) for the REF (blue color) and AR (green color) scenarios. The thick lines represent the ensemble mean for each scenario. The bright green shaded region indicates that there is no statistically significant difference between the two scenarios, while the purple shading suggests that a statistically significant difference exists (significance estimated as described in Methods). The percentile of temperature used is noted at the title of each panel. In a given year and for every ensemble member, the Xth percentile of temperature is obtained from the globally averaged daily temperature timeseries.

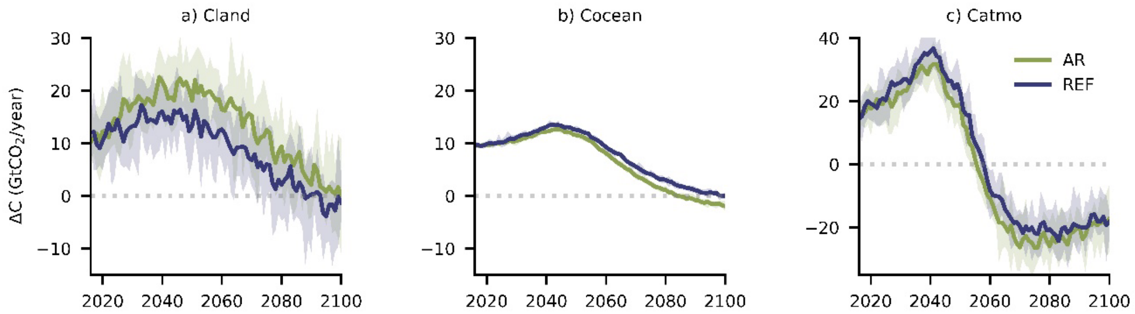

Supplementary Figure 4: Average yearly rates of change ($GtCO_2$/year) for the a) land (Cland), b) ocean (Cocean), and c) atmospheric (Catmo) carbon sinks. Mean quantities are represented with the thick lines, whereas shading represents the uncertainty range (minimum-maximum) for the AR (green) and REF (blue) simulations. For the land and the ocean positive values indicate an increase in carbon (sink behavior), while a transition to negative values indicates a transition to being a source (carbon release).

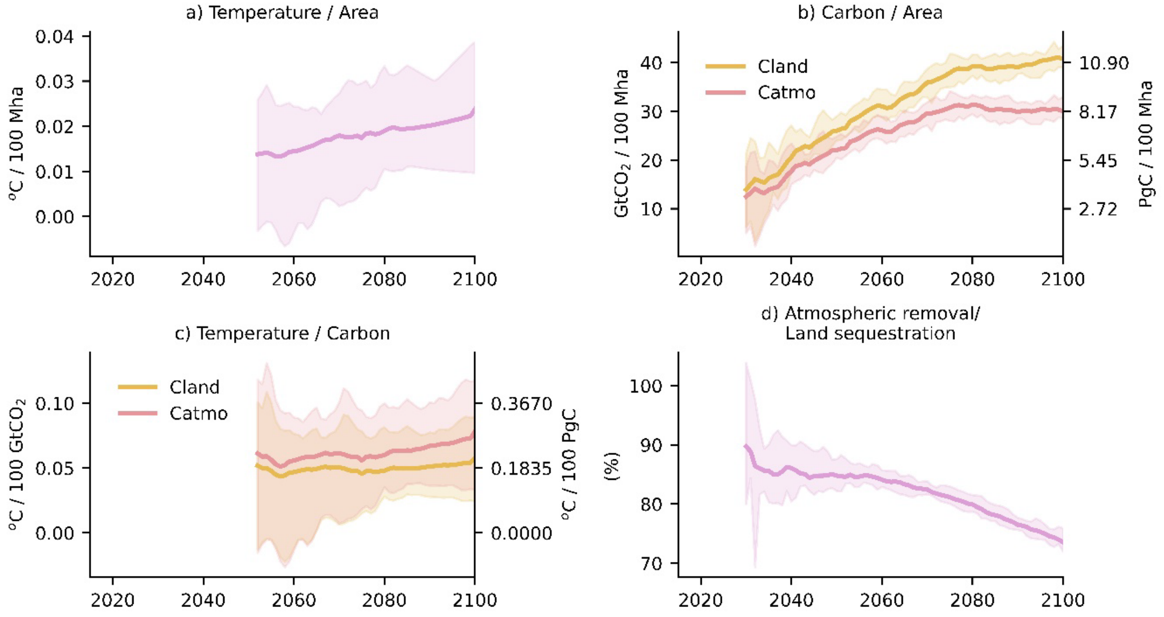

Supplementary Figure 5: Average yearly AR efficiencies represented here as: a) reduction of global average temperature per forest area increase (°C/100 Mha), b) atmospheric carbon removal and land sequestration per forest area increase ($GtCO_2$/100 Mha (left y axis), PgC/100 Mha (right y axis)), c) reduction of global average temperature per carbon removed from the atmosphere and sequestered in land (°C/ 100 $GtCO_2$ (left y axis), °C/100 PgC (right y axis)), and d) atmospheric carbon removal per carbon sequestered over land (%). Mean quantities are represented with the thick lines, whereas shading represents the uncertainty range (minimum-maximum). Temperature data are smoothed with a Savitzky-Golay [3] filter. Efficiencies with regards to temperature are shown from 2052 onwards, when the signal of temperature mitigation starts emerging, while from 2035 onwards for (b) and (d).

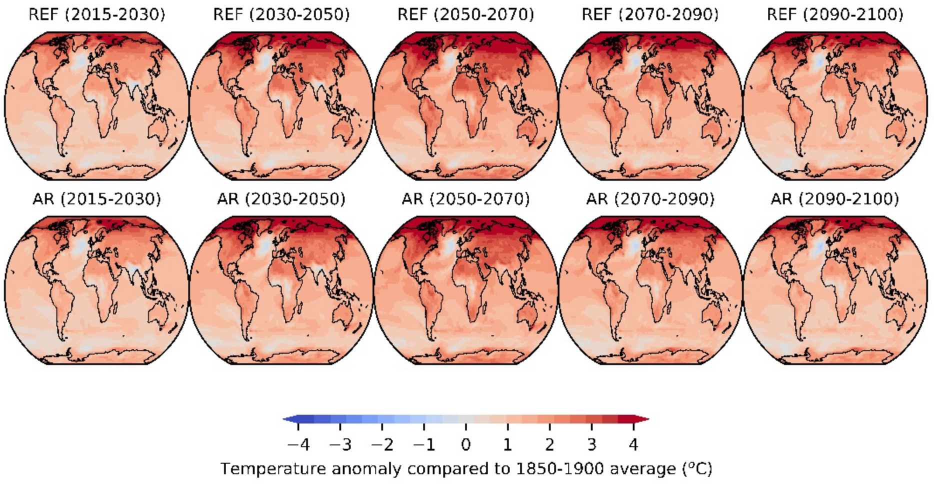

Supplementary Figure 6: Changes in temperature: The figure shows changes in mean 2m air temperature across time with respect to the 1850-1900 local average for the (top) REF, and (bottom) AR scenarios.

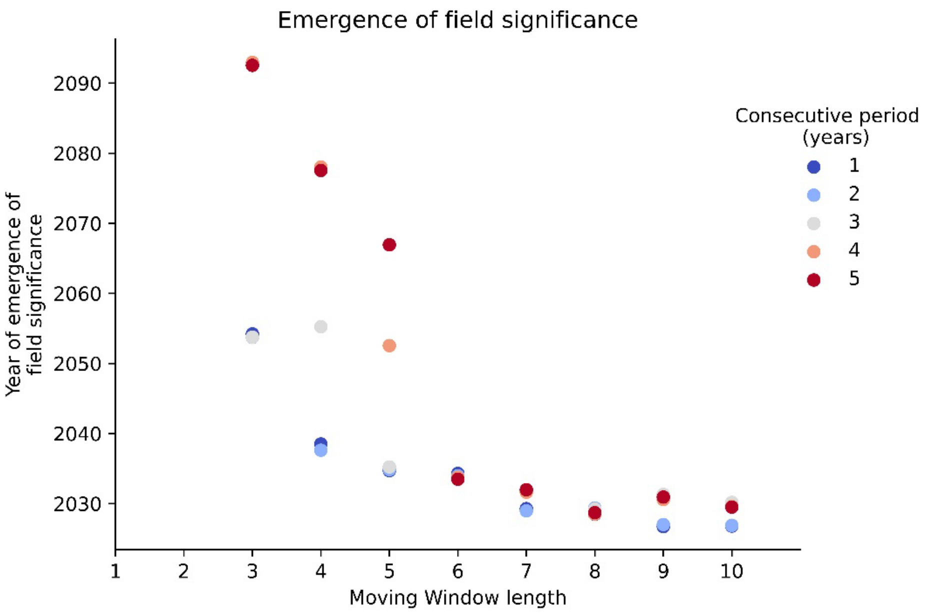

Supplementary Figure 7: Year of emergence of field significance (see Methods) (y axis) using the Bonferroni method [4], depending on the moving window length chosen (x axis), and the number of consecutive years rejecting the null hypothesis needed to declare significance (color). Missing (not plotted) values suggest that a signal has not emerged. Dots are slightly nudged across the vertical axis to aid interpretation.

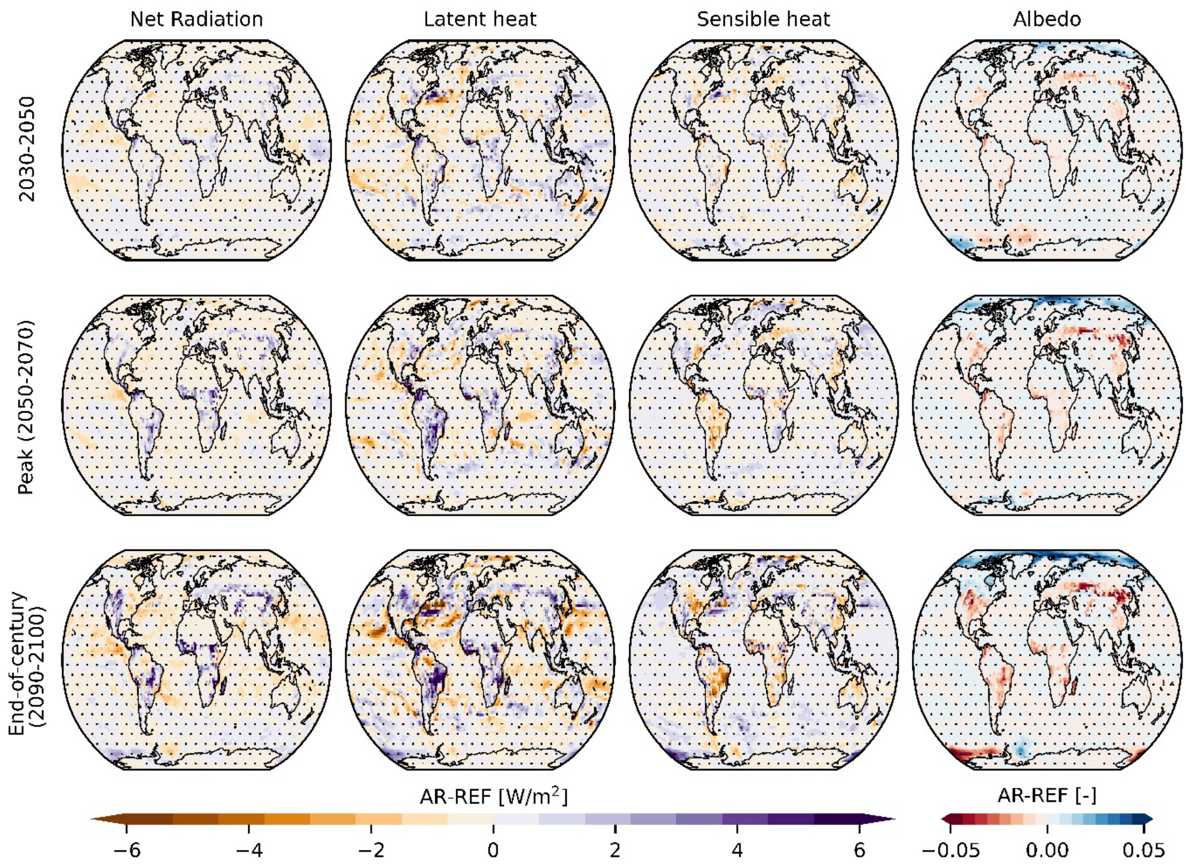

Supplementary Figure 8: Spatiotemporal pattern of changes in radiation and heat fluxes: From left to right, the differences in surface net radiation (W/m^2), latent and sensible heat fluxes (W/m^2), and albedo between AR and REF simulations during 2030-2050 (top), the period around peak warming (2050-2070) (mid), and end-of-century (2090-2100) (bottom) are shown. A negative value indicates a reduction in the AR scenario. Dots indicate regions where the difference is statistically insignificant at the 5% level, estimated with a two-tailed Student's t-test after correcting for lag-1 temporal autocorrelation [5] (see Methods).

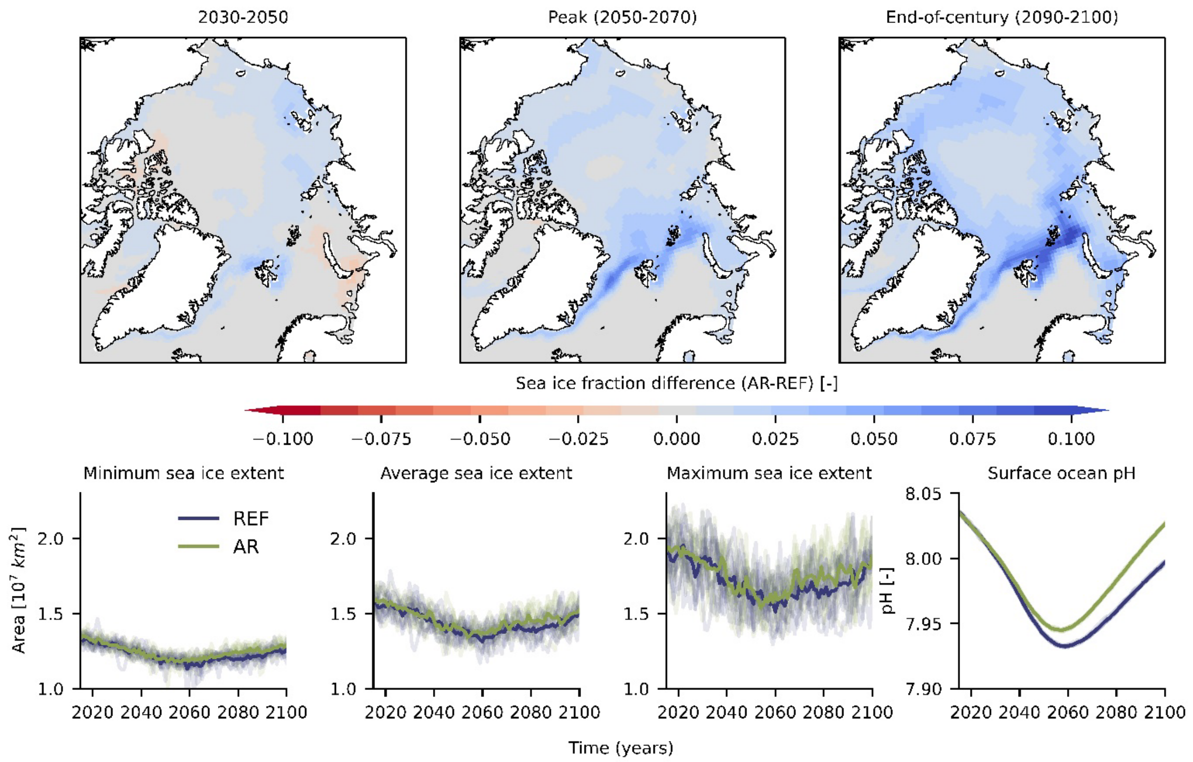

Supplementary Figure 9: Top row: From left to right, changes in sea ice fraction over the Arctic between AR and REF simulations during 2030-2050 , the period around peak warming (2050-2070), and end-of-century (2090-2100) are shown. Bottom row: From left to right, changes in global minimum, average, and maximum sea ice extent (10^7 km^2) for AR (green) and REF (blue) are shown, with the thick lines representing the ensemble mean for each scenario.

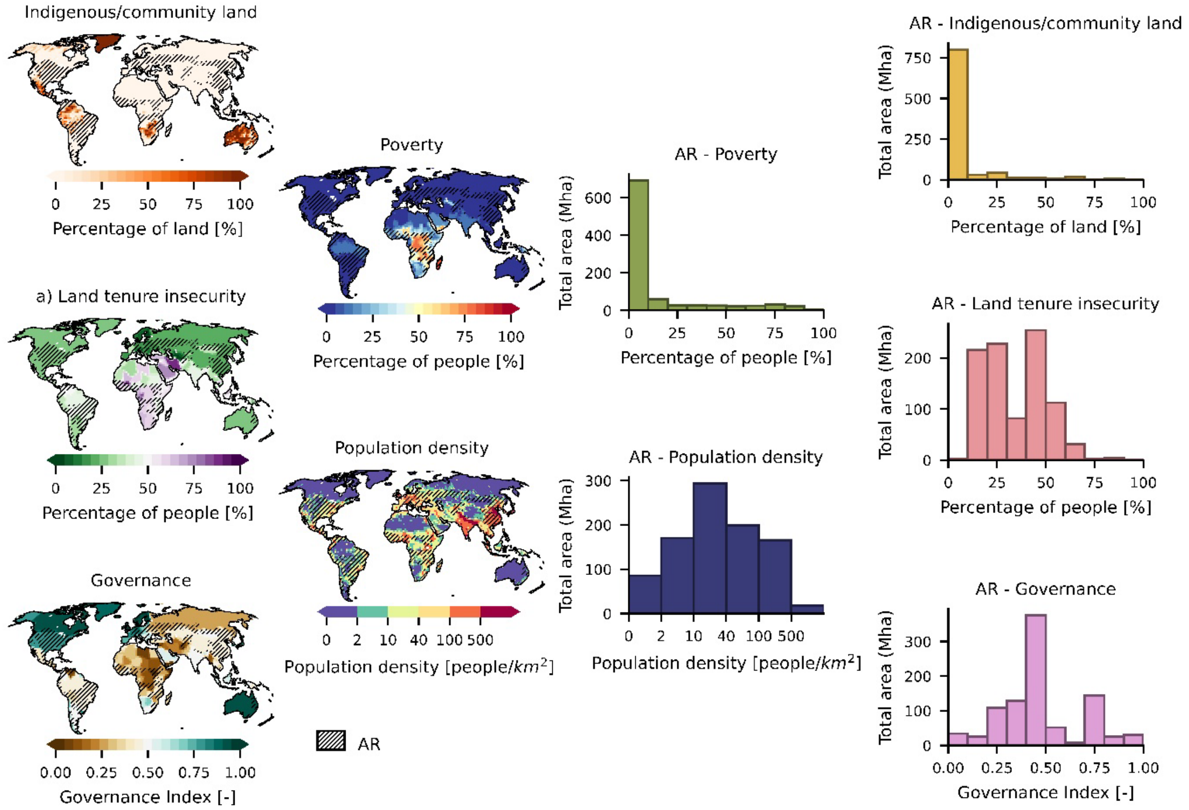

Supplementary Figure 10: Socioeconomic indicators: The maps indicate: a) indigenous and community land expressed as a percentage (%) of land per gridcell [6], b) land tenure insecurity expressed as the percentage (%) of people perceiving their land or property to be insecure [7,8], c) governance expressed with a composite governance indicator [9], d) poverty indicator

expressed as the percentage (%) of people below the $2.15 threshold [10], and e) population density (people/km2) [11]. The hatching shows the AR area in the employed scenario. The bar plots show the cumulative AR area (Mha) across different bins of the various socioeconomic indicators. Details on the socioeconomic indicators and their processing are presented in the Methods section.

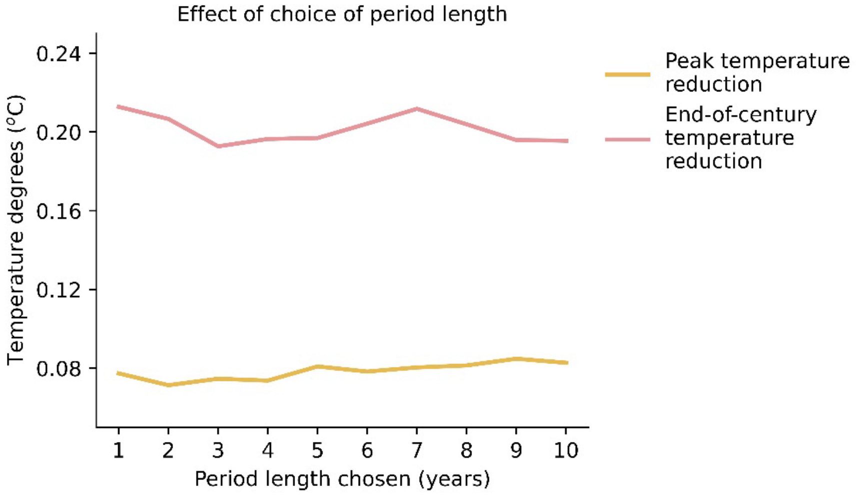

Supplementary Figure 11: The effect ot the choice of period length (x axis) in terms of estimating peak and end-of-century temperature mitigation between AR and REF is shown (see Methods).

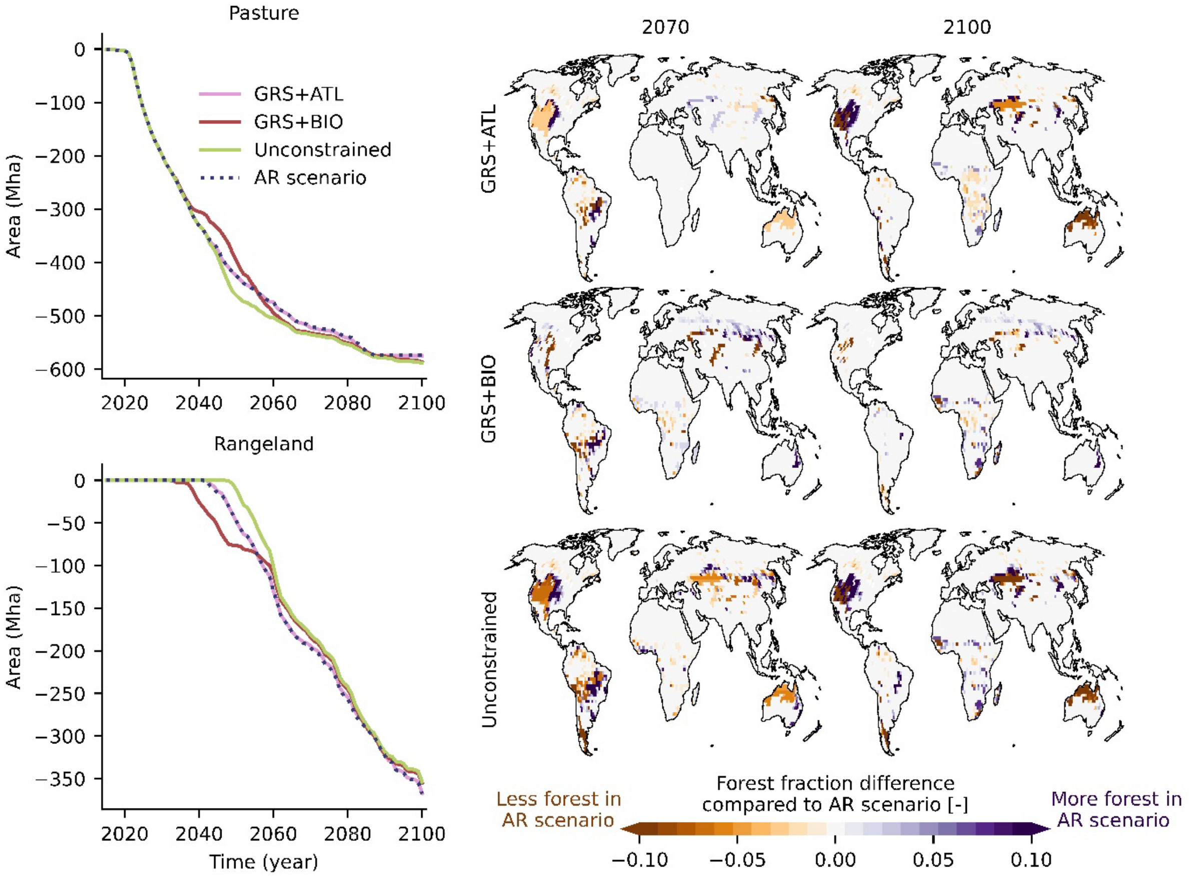

Supplementary Figure 12: The sensitivity of the AR scenario development algorithm to the different constraints employed (see Methods) is shown for configurations where the scenario is: a) constrained only by GRS and ATL (GRS+ATL), b) constrained only by GRS and biodiversity (GRS+BIO), and c) unconstrained. Left: Line plots show the changes in pasture (top) and rangeland (bottom) for the different scenarios, where also the scenario employed in this study is shown (noted as "AR scenario"). Maps: The maps show the difference in forest fraction between the different configurations tested here and the AR scenario employed in this study in 2070 and 2100.

Supplementary References

1. Dooley, K. *et al. The Land Gap Report 2022*. (2022).
2. Self, A., Burdon, R., Lewis, J., Riggs, P. & Dooley, K. *The Land Gap Report: 2023 Update*. (2023).
3. Savitzky, A. & Golay, M. J. Smoothing and differentiation of data by simplified least squares procedures. *Analytical chemistry* **36.8**, (1964).
4. Bonferroni, C. Teoria statistica delle classi e calcolo delle probabilita. *Pubblicazioni del R Istituto Superiore di Scienze Economiche e Commericiali di Firenze* **8**, 3–62 (1936).
5. Zwiers, F. W. & von Storch, H. Taking Serial Correlation into Account in Tests of the Mean. *Journal of Climate* **8**, 336–351 (1995).
6. Dubertret, F. & Alden Wily, L. Percent of Indigenous and Community Lands. Data file from LandMark: The Global Platform of Indigenous and Community Lands. Available at: www.landmarkmap.org. (2015).
7. Feyertag, J. *et al. Prindex Comparative Report: A Global Assessment of Perceived Tenure Security from 140 Countries*. https://www.prindex.net/reports/prindex-comparative-report-july-2020/ (2020).
8. Prindex. Prindex 2020 global dataset. (2020).
9. Kaufmann, D. & Kraay, A. Worldwide Governance Indicators, 2023 Update (www.govindicators.org). (2023).
10. World Bank. Geospatial Poverty Portal. World Bank Group. (2023).
11. WorldPop. WorldPop (www.worldpop.org - School of Geography and Environmental Science, University of Southampton; Department of Geography and Geosciences, University of Louisville; Departement de Geographie, Universite de Namur) and Center for International Earth Science Information Network (CIESIN), Columbia University). (2018).
