# Supplementary material for: Temperature overshoot responses to ambitious forestation in an Earth System Model
Source: Nat Commun. 2024 Sep 19;15:8235. doi: 10.1038/s41467-024-52508-x (PMC11413198; doi:10.1038/s41467-024-52508-x)
Supplement: Supplementary file 3 — Description of Additional Supplementary Files [file 41467_2024_52508_MOESM3_ESM.pdf]

**Description of Additional Supplementary Files**

**File Name: Supplementary Data 1**
**Description:** Excel spreadsheet that contains the detailed compiled list of past literature estimates on sequestration potentials.
